# Blood Letting

**Published:** 1837

**Authors:** 


### REVIEWS

AND

### BIBLIOGRAPHICAL NOTICES.

---

### Art. IV.—Blood-Letting.

*An account of the curative effects of the abstraction of blood; with the rules for employing both local and general Blood-letting in the treatment of diseases.* By James Wardrop, M. D., Surgeon to the late King, etc. etc. etc. London, 1835.

Such is the title of a small work, of 148 pages, which Dr. Wardrop has published on one of our most important therapeutical agents. It is a remarkable fact, that although blood-letting commands such an important station in our list of curative resources, yet that in neither of the works of Dr. Chapman or Dr. Eberle on Materia Medica and Therapeutics, is it at all distinctly treated. Among American physicians there is no one remedy of greater importance in the treatment of diseases than the lancet. In every part of our wide-spread country it is often resorted to—and no adequate substitute can be found for its vast remedial powers in many of the severe morbid affections which we are called day after day to treat.

We do not propose to enter upon any critical review of this work, but will endeavor to present its most striking facts and arguments.

Our author commences by General Observations on the Blood. Under this head the circulation, functions, living principle, morbid changes, quantity, sensible qualities, temperature, and coagulation of the blood are spoken of. There is nothing of interest under these respective heads, except under that of coagulation. The following are the remarks of Dr. W. on the coagulation of the blood.

"Coagulation is one of the most important properties of the blood. Whenever blood is removed from its proper vessels, a process of coagulation takes place, and this coagulation happens sooner or later, according to particular circumstances.

"Healthy blood coagulates in about three minutes and a half; the coagulation is usually completed in seven minutes, and in twelve minutes the mass becomes firm.

"Blood coagulates in the ratio of its specific gravity, the lighter the blood the more slowly does it coagulate; and coagulation takes place more or less quickly, according as the orifice from which it flows be small or large, or the stream fast or slow.

"Coagulation too is rendered slower by cold. We observe it take place quickly when the blood is received into a basin or flat vessel, and it coagulates soonest if the vessel be metallic. The rapidity of the coagulation also depends on whether it be the first or last portions which are abstracted, either from an artery or a vein.

"Blood, which is kept at rest, coagulates more slowly than when it is stirred or agitated. It coagulates most quickly when drawn through a small orifice, and allowed to trickle down the arm. Strong action of the arterial system appears also to dispose the blood to coagulate slowly, but, when the vascular action is diminished, as in fainting, coagulation takes place more quickly; and hence the assistance nature derives from syncope, in plugging up the orifice of a bleeding vessel.

"The power of the blood to coagulate is essential for the performance of many important functions in the animal economy, whether in a state of health or disease

"1st. It is this quality of the blood which arrests the bleeding from a wounded vessel.

2nd. Coagulation is the means of limiting spontaneous hemorrhages, by plugging up the open mouths of the vessels from which the blood is poured out.

"3rd. It serves to agglutinate the divided edges of wounded skin.

"4th. It is important in the restoration of a lost part by forming a covering to prevent the contact of the external air with the newly-exposed surface.

"5th. It forms a parenchyma or matrix for the passage of new vessels, in the restoration and regeneration of parts that have been destroyed, as in the process of granulation.

"6th. It is the power of coagulation which nature employs to prevent the bursting of an aneurysmal swelling.

"7th. And lastly, it is equally useful in plugging up the canals of diseased veins, and in agglutinating its wounds.

"As regards *spontaneous hemorrhage*, it has been already observed, that this coagulating power of the blood answers a very important purpose. There are many diseases wherein an effort is made by nature to relieve the general system, or a particular organ, of a superabundant quantity of blood, and little or no disposition is made to arrest its progress, until considerable debility or syncope supervenes, which has the effect of promoting the necessary coagulation. And, moreover, when blood is lost by a wound, the diminution in the quantity of the blood, by retarding the force of circulation, creates a power in that blood to coagulate more speedily, and thus arrests the hemorrhage. Hence is derived the useful practical lesson of encouraging a state of syncope, in order to assist in stopping hemorrhage. Indeed, when syncope does take place, during operations, we ought to watch the state of the wound after the syncope goes off before venturing to close it, for the vessels often begin to bleed, when, in order to prevent secondary hemorrhage, they ought to be secured by ligatures.

"The importance of this property of coagulation in the blood, is also exemplified in repairing injured or lost parts of the body. When the skin, for instance, is divided, the cut edges adhere, and this adhesion is effected by a quantity of coagulated blood being interposed between the lips of the wound. Here the coagulated blood seems to act as a mere bond of union, and I may observe, as a general rule, that whenever we are able to keep the lips of a wound together by the adhesive properties of the blood alone, blood is the most preferable, and the best plaster.

"Hunter believed that the coagulum thus formed between the edges of a wound possessed within itself a power of generating vessels, and thus became organised; but whether under the ordinary circumstances of reparation, new vessels are formed in the midst of the coagulum, or whether they shoot into the coagulum from the adjacent surfaces, and are continuations of those which have been divided, it is certain that vessels meet together and anastomose freely in the coagulum, which, along with the nerves that are also supplied, ultimately constitute a complete organization of what originally was a mass of coagulated blood.

The power of the blood to coagulate for the purpose of the restoration of parts, is exemplified when a portion of the skin is accidentally removed. In such a case, coagulated blood is first deposited ou the wounded surface, and coagulable lymph is then effused by the arteries between the coagulum and the wounded surface, for the purpose of forming a matrix for granulations; the growth of which granulations and their subsequent cicatrization repairing the lost part.

The power of coagulation in the blood is also employed by nature for the cure of *aneurysm*—one mode at least of spontaneous cure entirely depending on this process. When an aneuryism is formed, the blood which fills the dilated part of the vessel, or the proper aneurysmal tumour, does not circulate either in the same direction or with the same velocity as in the natural condition of the vessel. The consequence is, that a process of coagulation of the blood in the tumour commences, and the extent of this process varies according to the form, size, and position of the aneurysm. In some cases the tumour, however small, is found completely filled with a mass of coagulum, whilst in other cases the parietes of the artery have derived comparatively little additional thickness.

The coagulum, however it may be formed in aneurysmal tumours, is not produced quickly, and consists in the deposition of one lamina of fibrin upon another, which fortifies the parietes of the tumour, and prevents its bursting. In cases where the aneurismal cavity is completely filled, an absolute cure of the disease is thus accomplished, the diseased portion of the vessel being rendered no longer liable to rupture. There is, however, a difference to be observed between the coagulum of an aneurysm and a common clot of blood, though the distinction has not been clearly pointed out by pathologists.

"This quality of coagulation in the blood, which, as I have endeavored to show, is so powerful an agent in arresting hemorrhage as well as in accomplishing other restorative processes, has in some persons been found wanting, and hence bleedings even from very small vessels have not been capable of being stopped, and the hemorrhage has in some instances even proved fatal. As few such cases are recorded in medical works, it may be expedient on this occasion to bring together all the materials I have been able to collect on this interesting subject, and mention those examples of this peculiarity of the blood which have come more immediately within my own knowledge.

A gentleman found on several occasions great difficulty, and it often required many hours, before he could stop the bleeding occasioned even by superficial scratches, such as he sometimes met with in shaving. At length he accidentally received a slight wound on one of his fingers, and every effort to arrest the hemorrage failing, he expired.

"I attended a patient where the introduction of a common seton needle in the side was followed by a fatal hemorrhage. The gentleman who had an enlarged spleen, was advised to have a seton introduced, and the operation was performed in the usual manner by Sir Astley Cooper. Alarmed by the quantity of blood oozing from the wound, I was sent for to see the patient in the evening of the same day. On withdrawing the cord, pressure carefully applied with graduated compresses did not avail, and the hemorrhage being so profuse as to make it appear probable that some vessel of considerable size had been wounded, I thovght it expedient to divide that portion of integument between the two perforations made by the seton needle. Having done this, I found that the blood issued from numerous orifices, and I secured no less than nine vessels with ligatures. Blood continued, however, to ooze from numberless small orifices over the whole surface of the wound, which every mode of treatment usually resorted to failed in arresting, and the patient died in a few days.

"An interesting case which I conceive to have been of the same description, is recorded by Mr. Blagden in the eighth volume of the Transactions of the Medical and Surgical Society. A young man, at different periods of his life, experienced great difficulty in stopping the bleeding from very slight wounds. At length he had a tooth extracted, which was followed by a violent hemorrhage, that neither stypics, pressure, the actual cautery, nor a ligature on the carotid artery, could control, and he died in a few weeks after this injudicious operation.

"Mr. Wilson mentions a case where a child died from the bleeding of a small bite on the tongue. "I once had an opportunity of inspecting the body of a young person in whom, during life, a very small degree of pressure on any part of the skin, produced the appearance called black and blue to that degree, that the nurse in dressing him, although a very careful woman, frequently left the impression of her fingers on different parts of his body. I have frequently seen the child with bruises of a livid color, arising from falls, or even pressing against any thing with the slightest force; if a pin happened to scratch him, great difficulty occurred in stopping the bleeding, and the application of a single leech was productive of so much danger from the continuance of the hemorrhage, that the child's life was nearly lost. The complexion of the child was fair, and the appearance delicate, but not unhealthy; the pulse was full, but never hard, and the functions of the viscera generally seemed to go on as in other children. When between three and four years of age, the child bit its tongue; there was in impression of the teeth both on the upper and lower surface, but not very deep; the bleeding from the wounds continued for some hours, and having resisted all attempts to stop it, I was requested to see the child. I tried compression and every kind of styptic, which produced a temporary, but no permanent effect, so that although the child lived five days, and during that period I saw it several times in the day, and frequently remained an hour or two with it, though I never failed in producing a temporary stoppage of the bleeding, it was renewed almost as soon as I left the house. I included the whole of the bleeding surface in a ligature, which for a short time stopped hemorrhage, but ulceration very soon took place, and a fresh bleeding occurred; to use the needle was now impossible, as the least puncture produced a bleeding almost as violent as that from the wound. I destroyed the surface by caustic, but the eschar was soon thrown off, and the bleeding renewed; the child became very weak, and, as the bleeding occasionally occurred during sleep, was watched over very carefully; but on one occasion, when upon the supposition that he was asleep, he had not been looked at by the nurse for half an hour, she found that a very slight bleeding, not exceeding a teaspoonful, had taken place, and that the child was dead."

"A curious case came within my own observation, where this deficiency in the coagulating power of the blood appeared to be *hereditary*. A family of which there were several branches, all found a particular difficulty in stopping the hemorrhage from any very slight wound which they might have accidentally received, and one of the brothers who had a common operation for fistula performed, died of a bleeding which was occasioned by the incision made in the operation.

"But by far the most interesting case which has come within my knowledge, and where there was not only a want of power in the blood to coagulate, but where that peculiar state of the sanguineous fluid existed in many branches of the same family, occurred in the practice of Mr. Ward, surgeon, at Ewell, and I shall conclude this part of the subject by relating the circumstances as they were communicated by him to me:—

"'The particular state of the boy's constitution was manifested at two months old, when his arm was unusually bruised by a slight blow; and soon after this accident, there was much difficulty in restraining the hemorrhage occasioned by a superficial wound of the lip, and since then, whenever he has had a slight scratch. When he first came under my care, he had received a superficial wound in the palm of the hand a few days previous, and appeared much exhausted from hemorrhage. I attempted to restrain it by pressure, oil of turpentine,—caustic,—and by strong acetic acid; and after giving him some aperient medicine, he took the diluted sulphuric acid in large doses, and astringents. The case becoming very desperate, for the hemorrhage continued unabated for two days after I first saw him, I now ordered a dose of superacetate of lead with opium every three hours, when the hemorrhage soon abated, but whether from the effect of the medicine or not I was unable to determine. It appeared to me to have had a considerable influence. I formed some idea of the extent of the hemorrhage by having the arm placed above a plate, when twelve ounces of blood was collected in as many hours. This blood was not at all coagulated, and I could only observe clots when the blood was suffered to collect in folded linen, and then in very small quantities. The boy, after this attack, was completely exsanguined, suffered excessively with all the symptoms of protracted hemorrhage, and it was several months before he recovered.

"'Since that time he has been under my care for a wound on the head, the hemorrhage from which was restrained with less difficulty, because pressure could be more effectually used, and at the same time I applied the lunar caustic freely. The bandage remained for more than a week, when an ulcer, having a very offensive smell, of about the size of a shilling, remained. This went through the natural process of granulation, and certainly healed more rapidly than under common circumstances. At this time the boy is well, and is in the eighth year of his age.

"'The circumstances connected with this boy's family are equally remarkable. His mother has a numerous offspring, and a brother, who is twenty-two years old, is afflicted in a similar way, and is also an almost constant sufferer with rheumatic gout. He had five uncles and two aunts; all his uncles had the same hemorrhage tendency, three died from a division of the frænum linguæ, one from the extraction of a tooth, and the other had the same disease, but died from some other cause!

"'The aunts had not that tendency in their persons: the one had three boys, two of whom are thus afflicted; the other has two boys and two girls: both boys are afflicted in the same way.

"'From the above account, there can be no doubt of the *hereditary nature* of the disease; and it is a singular circumstance, that it is confined in this family to the male branches.'

"The treatment of such cases becomes an object of interesting inquiry, and a successful analysis of the blood might perhaps teach us how to supply the deficiency in its power of coagulation. Those substances, which dispose the blood to coagulate, seem to have more effect in restraining the bleeding for a time than those which excite the artery itself to contract. Hence styptic applications have been employed; and these act by producing a chemical change in the blood with which they are mixed, and thereby form a cake or cement, to supply the place of a coagulum.

"Sir C. Scudamore recommends with much confidence, the application of a saturated solution of the sulphate of alum for arresting hemorrhage. This salt, by producing an immediate coagulation of the blood, forms a coagulum at the mouth of a bleeding vessel, and thus accomplishes the first step of that process by which bleeding is arrested. The alum solution should be used warm. in which state it favors the coagulation, and this warm application is not to be considered inconsistent with the application of cold to the parts in the immediate vicinity of the bleeding vessels, the effects of which in diminishing the action of the vascular system is well established. The alum solution is to be applied either by a compress wet with it, moderately pressed on the bleeding part, or it may be injected into a bleeding cavity, such as the nose, uterus, bladder, or rectum.

"Besides the causes to which I have now alluded, wherein a want of the power of coagulation in the blood seems to depend on a peculiarity of constitution, and in some instances on a hereditary taint, there are other causes which produce déficiencies in the power of the blood to coagulate.

"In many diseases, where the solids are verging towards putrefaction, the disposition of the blood to form a firm coagulum is much lessened, as in typhus fever and scurvy. In animals, too, that die suddenly from over exertion, the blood does not coagulate. Most persons are aware of the difference in the blood of a hare which has been shot, and of one which has been run down by hounds; and in persons who are suddenly killed by lightning, or during violent fits of passion, or criminals by hanging, the blood does not coagulate. Alkalies, and common salt, entirely prevent the coagulation of the blood, whilst mineral acids promote that process. Is it on this principle that the internal use of mineral acids is so beneficial in hemorrhages? Can this effect of sea salt on the blood, account for the state of the sanguineous fluid in persons affected with sea scurvy? it being well known that this complaint is the effect of eating salt provisions, and that the blood in such persons is unusually thin. We know that this complaint is also cured by the exhibition of acids. It is a characteristic feature in the menstrual flux, that the discharge does not coagulate like common blood, nor does it ever become putrid, until after artificial evacuation, as is exemplified in cases of imperforated hymen, where its exit had been often prevented even to an advanced period of life.

"The consistence of the blood is another quality which merits attention. Blood, in inflammatory cases, is not only longer in coagulating, but it is also much thinner than natural, which thinness has been proved by Hewson to depend on the greater attenuation of the coagulable lymph or fibrin. The whole mass of inflamed blood, before coagulation, is actually thinner than the serum of the same blood after coagulation has taken place, and the crassamentum or clot separated from it. Hence the disposition of arteries in an inflamed part to throw out coagulable lymph, may, more or less, depend on this change in the blood.

"Whilst blood is flowing into a basin, our eye enables us to form some notion of its consistence, but this is best ascertained by the time it requires to coagulate. A firm texture of blood has been generally considered as a mark of strong action in the arteries, and as pointing out the propriety of abstracting it, and, on the other hand, when its texture is very loose, the utility of repeating venesection has been considered questionable.

"Soon after coagulation, blood separates into two substances, the crassamentum and the serum.

"The serum, or watery part, exudes through the pores of the coagulum, whilst the crassamentum contracts, leaving the sides of the vessel containing it, and preserving its form.

"The proportions of the serum and crassamentum are various. In healthy blood they are nearly equal quantities; whereas, in very stout and laborious people, and also during some inflammatory diseases, the crassamentum is in the larger proportion.

"The serum is a fluid of a pale straw colour, has a slight saline taste, is somewhat viscid, rather heavier than water, and mixes readily with it. When exposed to a moderate heat, it coagulates into a light straw colored glutinous mass, and the same effect is rapidly produced by its sudden admixture with an equal quantity of boiling water.

"The part of the serum which is thus coagulated, either by the application of heat, or an admixture of acids, possesses most of the chemical properties of the white of egg, and has been termed the *albumen.*

"When serum is coagulated by heat, there oozes from the mass a small quantity of a viscid substance, which has been termed the *serosity*, and is a mucous fluid.

"The serum has been found of a white color, like cream, and to contain globules, which peculiar appearance has been attributed to an admixture of the chyle with the blood. This appearance, however, has usually been met with in the serum of persons whose appetite has been impaired, and who have been subject to sickness and vomiting. Hewson supposed this change of color to arise from absorbed fat, and says that all the persons in whom he found the serum white were plethoric, and were relieved by blood-letting. If a person be bled soon after dinner, the serum appears milky.

"The serum is in greater quantity in what has been called *sizy* than it is in healthy blood, and the greater quantity which makes its appearance when you examine a vessel containing such blood, arises from the strong contraction of the fibrin forcing all the serum out of the clot. In such blood, the coagulum is remarkable in its shape; in place of having a smooth, plane surface, its edges are inverted.

"When serum collects in a preternatural quantity, either in the cellular texture, or in any of the serous cavities, as those of the pleura, pericardium, or peritoneum, it produces the disease called *dropsy.*

"The red globules were supposed by Hunter to be an indication of physical strength; the stronger an animal was, the more red globules did its blood contain. Wild animals have a greater proportion of red globules in their blood than those which have been domesticated, and there is a curious difference in the colour of certain muscles in the same animal, depending, no doubt, on the colour of the blood; this is particularly remarkable in the pectoral muscles of the black-cock, one set being much paler than the others.

The colour of the globules themselves varies in different vessels of the body, and and there is a difference in the shade or intensity of the colour of the blood from the quantity of globules which it contains. There are also diversities of shade of the red in the different systems of vessels. It is of a bright scarlet colour in the systemic arteries, and purple in the veins, and it is changed from the scarlet to the purple in passing from the ramifications of the aorta into the veins. Hence the variety in the shades of red, in different inflammations, arising from the differences in the proportion of the number of arteries and veins of the inflamed part.

When kept at rest, the blood in the arteries becomes of a dark colour, and in extravasations of arterial blood into cavities, if not exposed to the atmospheric air, it also assumes a dark colour.

Although blood is usually dark in the veins, it is not so under all circumstances. For when a large orifice has been made in a cutaneous vein, the blood which immediately follows the withdrawal of the lancet is black, and this is sometimes succeeded by blood of a florid scarlet colour.

"The blood is also found of a florid colour in the veins of a part affected with common inflammation; and if the bandage used in phlebotomy has been kept some time on the arm, the blood which first flows from the orifice is very dark.

"In diseases obstructing respiration, the blood is likewise of a very dark colour. This is the case during a fit of asthma. In diseases where the respiration is quick, as in phthisis, the blood is usually florid, and indeed, in inflammatory diseases generally, the blood is more than naturally florid.

"The red globules are not distributed, like other parts of the blood, throughout the whole frame, as appears from the want of the red colour in certain parts of the body. In some diseases their number is increased, at least in particular parts, and this may alone depend on the change in the size of the containing vessels. When much blood has been taken from the body, the red globules are not so soon renewed as in other parts, hence the paleness which often exists for a long time after copious depletions.

"Mr. Brande found, that the menstrual flux in its component parts resembles a very concentrated solution of colouring matter in a diluted serum.

"Changes are also observed in the colour of the external or cutaneous blood-vessels in many diseases, which informs us that changes must have taken place in the sanguineous fluid. Observe the varieties of red in the colour of the lips and cheeks, in the eyes, and in the skin covering diseased parts. Observe the various shades of redness in the skin around different ulcers—changes which alone, in many instances, tell us the nature of the disease!

"Richerand amputated the arm of a very old man, who for thirty years had an ulcer, which was connected with a varicose state of the veins of the limb; and the health of this individual was much impaired. During the operation it was observed, that "the blood as it flowed from the arteries was much less red than that which was lost by a young man whose leg was removed on the same day. In fact the venous blood appeared as if dissolved, of a violet colour, or somewhat like that produced by log-wood. It did not coagulate like that of the young patient, but separated into a serous fluid, containing a few clots very little colored.

"The coagulable lymph, *gluten*, or *fibrin*, is that part of the blood which, as has already been mentioned, forms with the red globules the *crassamentum* or clot.

"When the crassamentum is deprived of its red globules which may be readily done by allowing a small stream of water to pass upon it, and by squeezing out the serum, what remains is the coagulable lymph. It is a white, tough, elastic mass, having a fibrous appearance, even in some instances apparently formed into laminæ.

"This is the ingredient of the blood which renders it susceptible of coagulation, when removed from the living body, both in healthy and in certain diseased processes,—a change, which, whether we regard its rapidity, its degree, or its various modifications, forms an important diagnostic character in particular diseases.

Mr. Dowler, in his ingenious paper, observed, that when inflammation takes place accompanied with effusion, the first substance that escapes is serum,—but if the inflammatory action increases, *fibrin* is effused, and the inflammation still increasing, pus is then formed.

" While the blood is in a fluid state, the coagulable lymph cannot be separated from the serum, but in disease this separation often takes place. Hence coagulable lymph is effused on the surfaces of inflamed membranes, or into cavities, such as those of the pleura and peritoneum; whilst in other diseases, the serum is separated from the blood, and collected in cavities, forming the different dropsies.

"The coagulable lymph is found in the greatest quantity in the blood of those afflicted with inflammatory affections of the fibrous textures, particularly in rheumatism.

" There is a very considerable difference to be sometimes observed in the quantity of the coagulable lymph in blood taken in different cups from the same patient at the same bleeding. In some instances this difference has been observed nearly one-half. The coagulable lymph of healthy blood is more firm than that of sizy blood.

" Whilst the blood is coagulating, the process takes place so quickly in healthy blood, that the red globules are diffused throughout the whole of the crassamentum. But this is not the case in some diseases wherein the coagulation occurring more slowly, the red globules, from their greater specific gravity separating from the coagulable lymph, are found disentangled at the bottom of the vessel. In some cases the coagulation takes place when the red globules have scarcely reached below the surface of the crassamentum ; and it is this portion, deprived of red globules, which forms what is called the " buffy coat."

" The buffy coat or " inflammatory crust," as it is likewise called, is of very different degrees of thickness, and the time for its production varies from a few minutes to upwards of half an hour.

" The appearance of a buffy coat is generally considered as one of the most striking characters of the inflammatory diathesis. A buffy coat, however, is not to be deemed, either as a certain test of inflammation, nor is it a safe index of the propriety of blood-letting. Whilst inflammatory diseases are at their acme, there is often no appearance of the buffy coat in blood taken from a vein ; but in the latter stage of inflammatory disease, and when blood can no longer with propriety be abstracted, the buffy coat becomes remarkable. In local inflammation, before the constitution appears much influenced, the blood which is drawn from the larger vessels of the inflamed part, has been found to form a buffy coat, whilst blood drawn from distant vessels does not show the same disposition.

" Dr. Tweedle once saw the buffy coat in blood taken from the temporal artery of a person labouring under pneumonia, and the great prevalence of the buffy coat in diabetes first lead Dr. Watt of Glasgow to treat that disease by frequent bleedings.

" In the advanced stages of pregnancy, it has often been observed, that the blood has a peculiar disposition to form a buffy coat.

" Dr. Hamilton has observed that the blood drawn from the arm of the most delicate and debilitated individual, subjected to a course of mercury, exhibits the same buffy coat as blood drawn from a person labouring under pleurisy.

" If a man in health be bled immediately after taking violent exercise, the blood shows a buffy coat."

Under the next head we have the "utility of blood-letting—difference of abstracting arterial and venous blood—of a local abstraction of blood," etc.

The antiquity of the remedy is adverted to—which we look upon as a very idle matter in a small practical treatise on the remedy. The author speaking of arteriotomy, observes,—

" The result of my own experience is unfavorable to arteriotomy ; having generally found that the inflammatory symptoms recur much more frequently after a certain quantity of blood has been removed from an artery, than if an equal quantity had been taken from a vein; and I know that this coincides with the experience of others. There are, however, cases where the difficulty or even the impossibility of procuring the requisite quantity of blood from a vein, renders arteriotomy an important and even an indispensable operation for the abstraction of blood.

" A little reflection on these two modes of Blood-letting, may to a certain degree explain how this difference of effect is produced

" When blood is abstracted from an artery, there is an immediate diminution in the supply of blood to the part nourished by that artery ; but such is the vigour of the anastomosing branches, that the supply of blood which is thus cut off is very quickly restored. This is confirmed by observation. If one of the carotids be tied, almost immediately the temporal and occipital branches of the carotid of the opposite side can be distinguished through the integuments, dilating themselves, becoming tortuous, and struggling, as it were, to circulate an additional quantity of blood. I was first led to make this remark in the case of a child, on whose carotid artery I had placed a ligature for the cure of a large nævus on the cheek. Almost immediately after the operation, I observed the frontal, temporal and occipital arteries of the opposite side of the head enlarging, increasing in their action, becoming tortuous, and actively employed in supplying the place of those vessels whose channels had been obstructed. Indeed it was a knowledge of this function of the anastomosing branches of arteries, when a trunk is obliterated, that led Hunter to perform the " high operation," for popliteal aneurysm.

" The same interesting phenomenon is exhibited in the eye. If an artery on the sclerotic conjunctiva, passing into the speck of the cornea, be cut through, vessels containing red blood will be immediately perceived, stretching across the cornea from the opposite side, to supply the place of the vessel which had been divided.

" Whenever the supply of blood to a part is diminished, the arterial system, by a wise provision of nature, makes an effort to throw blood by another channel to the part which has been deprived of its natural quantity of blood, and the action of the heart and arteries must be more or less increased; but the abstraction of blood is followed by no such increased action of the arterial system,—there is no local diminution in the supply of arterial blood,—no effort made by the arteries to supply the place of the venous blood which has heen removed. On the contrary, a diminution in the supply of blood to the heart by the veins will, as I have just observed, have the effect of diminishing the vigour of the heart's action, and consequently, that also of all the arterial system.

" The effects of opening the temporal artery in puriform opthalmia, will illustrate the differences between arterial and venous depletion in the treatment of inflammation. An eye injected with red vessels, will be suddenly relieved by opening the temporal artery, and the conjunctiva will become quite pale, inducing us to suppose that the inflammation is subdued. I have generally found, however, that sooner or later, even in a few hours, the inflammation returns ; and others have made the same remark: whereas, if a vein in the arm be opened, and blood taken to the full extent, the good effects of such depletion are permanent.

"There is, therefore, a distinct and important difference between the changes which take place in the action of the vessels of a part, when blood has been taken from an artery or from a vein, and it explains the difference in the effects which I have always found when those two models of abstracting blood have been employed.

" It is also extremely probable, that there will be a variation produced according to the kind of blood which is taken away,—that the removal of a pint of arterial blood will produce a different effect on the system, from the removal of the same quantity of venous blood. What these differences are I cannot pretend to specify, but one proof that the abstraction of venous blood is the more useful in the cure of disease is, that in those natural or " spontaneous hemorrhages" which are so salutary, such as those from the nose and hemorrhoidal vessels, the blood which is discharged appears to be chiefly venous. Where leeches and cupping are had recourse to, they no doubt remove both arterial and venous blood at the same time; so that the circumstances under which these operations are performed, do not furnish us with any opportunity of discriminating between the comparative effects of the abstraction of venous and arterial blood.

"Another point which ought to be borne in mind, when weighing the advantages of abstracting arterial and venous blood, is, that the temporal arteries as they pass over the zygoma of the temporal bone, can only be once opened at the proper point for the operation. For arteriotomy is not like venesection,—the wound made in the vein when united, leaves the canal of the vessel entire; whereas when an artery is opened, it is requisite, after taking the necessary quantity of blood, to divide the vessel completely in order to allow it to retract, that the bleeding may be stopped; for it is impossible to attempt this by compression alone. The canal of the artery becomes, therefore, by the operation of arteriotomy, completely obliterated, and as the circulation is afterwards carried on by a greater or less number of enlarged anastomosing branches, neither the original trunk nor a branch of sufficient calibre will be found in the temporal region, on which the operation of arteriotomy can be repeated, should such a measure be ever deemed necessary."

The question, which is one of importance to the physician in inflammatory diseases, is, shall I bleed generally, or locally—or both? General bleeding where there is much general disturbance of the circulation should always precede local detraction of blood.

We accomplish two objects by general blood-letting. 1st. We diminish the momentum of the heart's action to such an extent that when we employ topical bleeding, the capillary vessels of the part, being thus depleted, are not rapidly re-filled, which would be the case if the urgent pumping force of a powerful venticular contraction was maintained. 2d. We, by general blood-letting, so diminish the impulsive energy of the heart as to render it expedient to take by local bleeding only a comparatively small amount of blood. The following suggestions in reference to local bleeding we think well worth extracting.

"The effect of losing a small quantity of blood locally, is, indeed, often surprising. Of this I might give some remarkable illustrations. An instance of the kind to which I allude, occurred in a lady, who, after employing local and general bleeding for a disorder in her head, applied to an itinerant doctor, celebrated for curing headachs. He introduced an instrument into the nose, by which he wounded the Schneiderian membrane, and produced a flow of blood; and the evacuation which followed, completely cured the headach. It was in a case of this kind that Galen is said to have acquired much renown, by successfully prognosticating that a patient would have a flow of blood from the nose, by which the affection of the head would be relieved.

"I had often thought that some mode might be contrived, by which an artificial flow of blood from the ethmoidal vessels could be produced,—as there are, no doubt, many affections of the head which would be relieved by such an operation, —or that leeches might be safely applied to the interior of the nostril.

"A lady had long suffered from headachs; I advised her to apply leaches within the nostril. One was accordingly applied on each side of the septum, and her headachs were completely relieved. Another lady, after severe mental affliction, complained of uneasiness in the head, for which blood-letting was employed. She had frequent returns of the headach, and was always relieved by depletion, leeches being sometimes applied behind the ear, on the temples, or on the feet.—On one occasion, when sense of fullness continued in the frontal region, after trying the usual modes, I advised her to apply a couple of leeches to the nasal septum, and the bleeding had the happy effect of completely relieving her head. Subsequent to that period, she had occasional returns of headach, and she found that a small bleeding from the nose never failed to give a more decided relief than she had ever obtained from any other mode of blood-letting.

"The practice of applying leeches within the nose is well exemplified in a patient who was under the care of Mr. Miller of Enfield "A gentleman, fifty-six years of age, had for several weeks complained of a sense of weight and pain, extending along the forehead, particularly in the region of the frontal sinuses; purgatives, and leeches to both temples, were resorted to with little benefit, which led me to adopt the practice lately recommended by Mr. Wardrop, of withdrawing blood from the vessels of the Schneiderian membrane. I applied one leech within each nostril, which bled freely, and I had the satisfaction to find the patient next day, completely cured."

"I have now had frequent opportunities of testing the comparative advantages of the different modes of abstracting blood in affections of the head, and I am fully persuaded that the application of a small number of leeches, and these, on the lining membrane of the septum of the nose, is, in many cases, the most preferable mode of blood-letting, more particularly in those where the uneasiness in the head is limited to the frontal region; and also in cases of chronic inflammation of the eye, and inflammatory affections of the lachrymal sac. In all such cases I have repeatedly recommended patients to apply the leeches alternately on the nasal septum, and on the part adjoining the inflamed organ, and to compare the effects of each; the result of this has almost invariably been, that they have given a decided preference to the abstraction of blood from the nose."

The practice of Mr. Hutchinson, of free scarification by incisions in erysipelas, is commended highly by Dr. W.

Mr. Travers, we observe, in his late work on Constitutional Irritation, the second part of the work published ten years ago, warmly eulogizes this mode of treating erysipelatous inflammation.

When is general blood-letting indicated? In answer to this inquiry, the author remarks—

"The leading symptom by which the constitutional disturbance demanding venesection is indicated, will be found in the *quality of the pulse;* and, in deciding on the propriety of blood-letting, it is not always necessary that we should find the pulse frequent, or tense, or full, or hard, but to be on all occasions satisfied of the propriety of abstracting blood from a vein, whenever, in any complaint of an inflammatory type, or under circumstances such as those which often arise after operations, or from accidents, there is the least deviation in the pulse from the natural standard. No sooner have a few ounces of blood been abstracted in such cases, than the pulse will be observed to "rise," as it is called, and to acquire volume and power; thus indicating the propriety of the measure.

"There is nothing more uncertain than trusting to the qualities of the pulse which are usually described as those which alone indicate the propriety of blood-letting; and we ought ever to be aware of the important fact, that, during an attack of inflammation, the pulse varies according to the organ which is affected, and is even different in inflammatory affections of different textures of the same organ,—except in one particular character, which shall be presently pointed out.

"The mere increase in the *frequency* of the pulse is a circumstance of little importance in estimating the propriety of blood-letring, compared with certain changes in its qualities; and, therefore, the usual practice of counting merely the number of pulsations, is apt to lead to an erroneous conclusion. In two patients, in each of whom the pulse beats 120 in a minute, the one may require to be plentifully bled, whilst the other ought to have cordials!

"Little difficulty occurs in distinguishing those states of the system which require general bleeding when their symptoms and character have become distinctly developed. It is before they arrive at this period, and when medical treatment is most available, that their true nature is apt to escape detection, and there are many states of disease where substantial advantage is to be derived from venesection, but where, at the same time, it could not, perhaps, be affirmed, from the presence of any particular symptom, that inflammation did actually exist. "Inflammation in some textures of the body," observes an intelligent writer, "is so obscure in the beginning, so insidious in its progress, and so rapid in its termination, that in all cases in which the surgeon hesitates respecting the necessity of bleeding, it is a wise plan not to deprive the patient of the benefit of the doubt, but immediately to proceed to venesection."

"It is certain that there is a period of disease, when bleeding is not only unnecessary but improper; and there is likewise a moment when venesection may be had recourse to with more advantage than at any other. To detect this precise period must, therefore, be of great practical importance. In those who have been wounded, or who have met with a severe accident, or on whom an operation has been performed,—in all such cases the action of the heart and arteries, which is at first diminished by the general shock received by the whole system, should be allowed to recover, or reaction should take place before resorting to blood-letting; and this rule applies generally to all inflammatory diseases. The chill, for instance, which a person receives from the sudden exposure to a change of temperature, and which lays the foundation of an inflammation in some of the internal viscera, produce a collapse of the whole frame, which is not followed, until after a certain period, by those inflammatory symptoms which require blood-letting.

"The peculiar feeling in the pulse to which I have alluded, and which points out, almost universally, the propriety of venesection, is sufficiently well indicated by the term "incompressibility." An incompressible state of the pulse may, I venture to say, be considered as pathognomic of inflammation, in whatever organ or texture that inflammation exist. I have already remarked, that each of the different textures of an organ, when inflamed, is attended with some peculiarity of the pulse. Inflammation of the dura mater,—of the cerebral pulp,—of the pleura,—of the mucous membrane of the bronchi,—of the pericardium,—of the muscular parietes of the heart,—of the serous membrane which lines its cavities,—of the peritoneal and mucons coats of the intestinal canal,—are each accompanied by peculiarities of pulse; but in all we can detect an incompressibility. For whether the pulse be full or small, hard or soft, frequent or slow, if with the point of one finger the artery be pressed at the wrist, we shall perceive with another finger applied to the artery beyond, and at the distal side of the first finger, that unless a very considerable degree of pressure be employed, the pulsation of the radial artery will not be destroyed, but the sensation, as if of a fine thread, or hair, will remain.

"This incompressible state of the pulse. indicative of the propriety of blood-letting, is illustrated in some of the subsequent cases; and we may often observe instances of persons being relieved from internal disease by profuse and spontaneous hemorrhage, and in whom no deviation in the pulse, from its natural state, could be detected, except as regards this feeling of incompressibility.

"Attention to this quality of pulse I can with confidence assert has scarcely ever failed to guide me, in the use of phlebotomy, even in otherwise doubtful circumstances; and a conviction of the accuracy of the observation has led me to adopt bleeding in many cases, where I could not otherwise have ventured to employ it, and where there were not present any of the ordinary indications of inflammatory action. It was this state of pulse which first pointed out to me the propriety of employing venesection in erysipelas,—in cases of sloughing ulcers,—in exanthematous fevers,—in cases of poisoned wounds,—in specific diseases,—and in certain stages of several other affections afterwards to be noticed, and where an opposite practice is frequently, nay usually, followed.

"In most of the diseases which have now been alluded to, by a superficial examination of the pulse, an impression is generally received that there is a feeble state of the system, and hence, in place of a depletive or antiphlogistic mode of treatment, wine, bark, and stimulating remedies, have been employed. But we can be readily satisfied of the result of these two very opposite modes of treatment in some of those diseases, by observing the practice in the different hospitals of this city, where patients, afflicted with similar ailments, are bled profusely by one practitioner, and by another, get as much bark and wine as their stomachs can receive.

"Though I have never observed an incompressible state of the pulse, without having found venesection to be an expedient measure, yet there are other qualities perceptible in the pulse which also indicate the propriety of blood-letting. A hard pulse,—a firm pulse,—a full pulse,—a wiry pulse,—an oppressed pulse, all, in their turn, either taken singly, or combined with other symptoms, more particularly local pain, hot skin, and white tongue, point out the propriety of employing venesection.

"No power of discrimination requires more attention and experience than such a knowledge of the pulse as enables us to distinguish its various peculiarities, both in health and in disease. This knowledge entirely depends on the exercise and improvement of the sense of touch. Like that of sight and hearing, the sense of touch differs exceedingly in acuteness in different persons, but in all it is capable of improvement. We must not expect at first to be able to distinguish these nicer differences in pulses which are palpable to an experienced finger, any more than we could expect at once to be able to perceive the slighter varieties in the shades of colour, so easily discernible by a skilful painter. All the senses require tuition. We ought to practise, by feeling a number of different pulses, both of people in health, and of those with disease, one after another; and by first observing strong contrasts, we ultimately become familiar with lesser differences in the pulse, all of which essentially assist in pointing out particular states of the vascular system."

We acknowledge our astonishment at two assertions of the author—"that there is frequently a difference in the frequency of the pulse of the two arms of the same person;" and "that the pulsations of the heart and arteries do not always correspond." In neither of these positions can we coincide—nor do we perceive on what data they are founded.

Pain is frequently a criterion of the necessity of sanguineous depletion. When the pulse is *weak*, but *hard*, the intensity of the pain should determine us to draw blood.

The importance of the first bleeding is thus discussed by the Doctor.

"There is no maxim of the practical correctness and importance of which I am more fully convinced than that the loss of a certain quantity of blood at the *first bleeding*, is of greater utility in stopping the progress of inflammatory diseases when general bleeding is required, than the abstraction even of a much larger quantity of blood, say sixty ounces, at three successive bleedings of twenty ounces each,—performed within thirty-six hours,—an infinitely greater degree of relief will be derived from taking two-thirds of that quantity at one bleeding. Not only will the progress of the disease be thus more quickly and decidedly checked, but the patient will be saved the loss of twenty ounces of blood.

"The good effect of abstracting so large a quantity of blood at the first bleeding might be expected, without adopting any preconceived opinions or theory on the subject; for, as we have been taught by experience, that the great benefit of abstracting blood is derived from the sudden change which such depletion produces on the action of the heart and arteries, it is evident that such change must be effected in a more decided manner by abstracting a quantity of blood at one bleeding suddenly, than by taking away an equal quantity slowly, and at more or less distant intervals.

"In almost all cases where bleeding is employed, there is a disposition on the part both of the patient and bystanders, and often too of the medical attendant, to be as sparing as possible in the evacuation of blood; whereas in the generality of cases an effort, I am convinced, should be made rather to take away as much as the patient can bear, and not to desist until there be rational grounds to expect that a second bleeding will not be necessary."

We think with the author that too much stress has been laid on the indication of syncope—and that an exaggerated view has been taken of the beneficent results of such a superinduced state.

"It has been laid down as a common rule for the treatment of inflammatory diseases, that blood should be abstracted until *syncope* is produced; by which we might be led to suppose that the fainting was either a certain token of the quantity of blood that should be taken away, or that it was the act of syncope itself, and not the loss of blood, which was to cure the disease.

"Hence we often hear of persons being bled until they fainted, as a proof of the vigour of the practice which had been employed, without considering whether the fainting had been produced by excessive depletion, or from the particular position of the patient whilst the operation was performed. Suppose two individuals similarly affected, and in every respect in the same state, one of whom is bled in the erect posture until he faints, whereas the other faints from the loss of blood whilst he is recumbent. In the first patient the syncope will probably be produced by losing one-half the quantity necessary to be removed before the second patient falls into a state of syncope.

"In having recourse to venesection, it is, therefore, important to consider whether the purpose of the operation be to abstract a certain quantity of blood from the system, or merely to produce syncope. When it is desirable to make a person faint with the loss of as little blood as possible, as, for instance, for facilitating the reduction of a strangulated hernia, or dislocated bone—then venesection ought to be performed in the erect posture. But for the abstraction of a certain quantity of the sanguineous fluid, in the treatment of diseases requiring depletion, fainting ought not to be considered as an index of the quantity of blood proper to be withdrawn; but the usual means should be taken to avert its occurrence, except when the patient is in a recumbent posture at the time the venesection is performed. Indeed when employing blood-letting for the cure of inflammatory diseases, we ought to be particularly cautioned against placing too much confidence in the idea that, if we have bled a patient *ad deliquium*, we have carried the bleeding to its full and necessary extent. On the contrary, when, in any case, syncope has followed the abstraction of an unusually small quantity of blood while the patient was in a recumbent position, then we ought, in one or two hours, again to examine the patient, when probably it will be found that the action of the vascular system has renewed its vigour, and the inflammatory symptoms continue, so that if blood be again allowed to flow from the vein, a very copious bleeding will sometimes be requisite to reduce the pulse.

"This "premature" state of syncope, as it may be designated, not only arises from the patient's being bled in the erect posture, but is sometimes the effect of a moral influence, and, therefore, when under such circumstances the fainting state goes off, the inflammatory symptoms may soon re-appear.

"A gentleman was seized with severe pains in the bowels, accompanied by a good deal of tenderness on slight pressure, along with some degree of febrile excitement. On opening a vein in the arm, only a few ounces of blood were removed, when the pulse sunk and he fainted. I visited him about two hours afterwards, and having recovered from the state of fainting, but not having experienced any relief, I again applied the bandage on his arm. Blood flowed freely from the wounded vein, and he did not fall again into a state of syncope, until he had lost about thirty ounces of blood; and this, along with purgatives, was followed with permanent relief."

We hear and read much of the changes which the blood undergoes in disease; and ever since the days of Hippocrates, the pathology of the fluids has been a favorite theme of declamation with the majority of the profession. We say, without meaning to give offence, declamation; for surely it is little better, as long as well ascertained and carefully reported facts are not adduced.

Now what are the different conditions of the blood which afford criteria for regulating the employment of the lancet? Dr. Wardrop thus writes:—

"There are appearances which the blood presents, both whilst it flows from a vein, and after it has been kept some time and allowed to coagulate, which appearances have been considered as affording criteria for regulating the quantity of blood to be withdrawn, and also for the repetition of the venesection.

"In violent inflammations, the blood usually flows from the wound in the vein with great force; whereas in other conditions of the system the stream is comparatively slow. In the first condition, bleeding ought to be as profuse, as in the latter it should be limited.

"The colour of the blood as it flows from the vein, also varies in different examples of disease, being in some very florid, or of a scarlet red, and in others of a crimson red or deep purple colour. The first state of the blood indicates the propriety of bleeding, whilst the latter points out a state of the system where but a very moderate depletion is admissible.

"The appearances which blood assumes after it has been allowed to stagnate, are also supposed to afford criteria for the repetition of venesection. The appearance of a buffy coat, and the comparative quantities of crassamentum and serum have especially been considered as an index of the existence of inflammation, and of the propriety of blood-letting.

"Whilst all these circumstances are mentioned, I must at the same time repeat what was stated in a former discourse, when explaining the different changes of the blood, that *these* can never be a criterion for estimating the quantity of the sanguineous fluid which it may be proper to remove, whilst the blood is flowing from the vein, neither can any appearance of the blood after its coagulation, guide us in repeating the venesection. The appearance of the buffy coat has been chiefly dwelt on as an important character of the inflammatory diathesis; but it has been already observed, that the buffy coat is not to be considered either as a certain test of inflammation, or as a safe index of the propriety of blood-letting. There is usually no appearance of the buffy coat in blood removed from persons affected with violent inflammations until the latter stage of the disease, and at the very period when the further abstraction of blood would be pernicious; in many diseases on the other hand where blood-letting is unnecessary or even hurtful, the buffy coat may be occasionally observed."

The author next speaks of the different methods of abstracting blood—these are familiar to the profession. We will notice one or two points more, and by additional quotations from the work enrich still more or pages—

Under "Injurious effects of Blood-letting, immediately after Injuries," etc., the author thus speaks:

"There is one class of cases in which blood-letting is often very unnecessarily, and sometimes perniciously employed. I allude to the common practice of bleeding persons immediately after an accident, or during an apoplectic or convulsive fit. In many accidents, more particularly where the head suffers, the first effects of the injury are a diminution or collapse of the vital powers; and, if under such circumstances blood be abstracted, a still further diminution of these powers is produced. Hence it is not until the powers of life have revived, or a *reaction* has taken place, that we should, after severe injuries, employ blood-letting.

"The servant of a medical society fell from a chair at the time of one of the meetings, and whilst still in a state of insensibility, surrounded with medical students she was bled. It was at least fifteen months after that bleeding, although it was very moderate in quantity, before she recovered her natural strength. Many have suffered, from a too early use of the lancet after accidents.

"In cases of *apoplexy*, I believe that blood-letting has frequently been carried to a very unwarrantable, and even fatal extent.

"In proportion to the violence of an apoplectic shock, so are the powers of life diminished; and hence, if the quantity of blood abstracted be regulated by the *severity* of the symptoms, in like proportion will the practice be hurtful by still farther diminishing the vital powers. When a person is in a state of insensibility from an apoplectic fit, those around are too apt to urge the necessity of bleeding, conceiving that the loss of blood will relieve the disease in the head.

"It is therefore proper in all cases to allow the *shock* to pass over and the circulation to revive before abstracting blood—and the quantity which is taken away ought always to depend on the vigour of the heart's action.

"A surgeon was sent for to see a patient, who had fallen down suddenly in an apoplectic fit; he immediately opened a vein, and after abstracting only a few ounces of blood, the pulse sank. When he visited this patient several hours afterwards, he found the powers of life so feeble that he ordered him cordials, by which the action of the heart, and arteries began to revive. Soon after this, however, a physician was consulted, and he *prescribed* a copious venesection, after which the patient sank rapidly, and in a few hours expired!

"In cases where there are organic changes in the brain's structure, and when the sudden apoplectic attack is caused by some vessel of the *diseased part* giving way and pouring out blood, blood-letting is of no avail, and when had recourse to when the pulse is feeble, and the vital powers already much diminished from the shock, it never fails to hasten the patient's death: therefore under such circumstanstances blood-letting ought to be resorted to with great caution.

"A lady considerably advanced in life was found during the night lying on the floor,—her servant, who was in an adjoining apartment, having been awoke by the noise of her mistress falling out of bed. She was found perfectly insensible, and the pulse so languid, that the surgeon who was sent for ventured to take only a few ounces of blood by cupping; her pulse never revived, and in four hours she expired. It was found that her death was occasioned by an effusion of blood into the ventricles, the heart and aorta being also diseased.

"It is in cases of this description that blood-letting, even to a small extent, must, by diminishing the powers of life, prove injurious, and, if carried beyond certain limits, inevitably hasten dissolution.

"A patient who had long suffered from an affection of the heart, and who had for many years complained of a pain in the occipital region, suddenly tumbled down senseless, and when he endeavored to rise, the left arm and leg had become quite motionless. On visiting him a few hours after this attack, I found his pulse so feeble and the powers of life so depressed, that in place of a depletive system of treatment, I recommended the use of ammonia, and camphor, -and what was remarkable in this case was that the paralized limbs gradually acquired their natural power, and in four days the paralytic affection was completely removed. Four months after this illness he was again suddenly attacked, and his condition being considered apoplectic, he was freely bled and purged, and in about twenty hours he expired!

"On opening the head a large clot of extravasated blood was found in the substance of the left hemisphere of the brain,—and in the right hemisphere, there was a large cavity which contained a small quantity of a dark coloured sanguineous fluid, not sufficient to fill it, and which no doubt was the remainder of the effusion which had taken place during the first attack. The arteries at the base of the brain were much dilated, and their coats considerably thickened. There was dilation of the left ventricle, its muscular parietes were thickened, and some portions of the semilunar valves were ossified."

The curative effects of abstracting blood in fevers; eruptive fevers—-erysipelas—acute inflammations—congestions—wounds and ulcers are discussed in the last chapter of the work.

The utility of bleeding in febrile affections is a familiar subject to American physicians. Eruptive fevers often demand the lancet. The author adverts to the employment of the remedy in them.

"Blood-letting may also be resorted to in the early stages of *scarlet fever*, particularly when there is a determination of blood to any particular part, as the chest or head. Sometimes the inflammation of the throat, the characteristic feature in this disease, is so severe, that much relief is obtained by local bleeding, more or less profuse; but when any of the vital organs are affected, then venesection is preferable.

"There is in this disease, as in erysipelas and other of the exanthemata, a notion with many, that patients cannot endure a depletive system, or that such is unnecessary for their treatment. It cannot be denied that, like most other diseases, the scarlet fever in many cases passes through a very mild course, having scarcely a symptom requiring any particular treatment; and there are examples wherein the pulse from the very commencement of the disease is feeble, and the powers of lsfe sink rapidly. We ought also to be aware, that cases of scarlatina do occur, where severe inflammatory symptoms come on, and of that degree wherein blood-letting is highly to be recommended. The additional vigour which the pulse acquires whilst the blood flows from the vein, will be an unerring guide for estimating the propriety of the measure, and the extent to which it should be carried.

"The benefit of blood-letting, when the febrile symptoms accompanying *small-pox* are severe, or when inflammation affects any particular organ, is equally great. An intelligent army-surgeon made it a general rule, from which he experienced the greatest benefit, always to bleed soldiers on the commencement of the fever preceding the eruption.

"A lady was attacked with severe febrile symptoms, for which she was profusely bled, and with great relief. On the following morning it appeared that she was afflicted with small-pox, and her medical attendant at first expressed his regret at having had recourse to venesection. Such, however, was the mild progress of the disease, when viewed in comparison with that of many who were affected in the same town, that all were convinced the blood-letting had been highly beneficial, and had mitigated the febrile symptoms.

"When considering the effects of local bleeding, I remarked that its benefits were exemplified very strikingly in cases of *erysipelas*, from the relief derived by the profuse hemorrhage which followed incisions made into the inflamed integuments. Such a practice, will, however, I am persuaded, be seldom found necessary, if blood-letting be had recourse to in the early stage of that disease.

"From a supposed *typhoid* character of erysipelas, it was, and with some still is, the general practice never to deplete or pursue an antiphlogistic plan of treatment; and I well remember visiting a lady who was suffering from a very severe attack of erysipelas in the face, and finding at her bed-side a large tumbler of wine-and-water, and that she was taking as much bark as her stomach could receive. By bleeding her freely at the arm, repeating the operation three times successively at short intervals, along with purging and antimonial medicines, she rapidly recovered; and another medical attendant expressed his surprise at the treatment I had employed, remarking that during a long attendance at a public hospital, he had never known blood-letting employed in erysipelas,—adding, that nearly all the cases which he had seen of that disease affecting the face and head had terminated fatally.

"In erysipelas there is usually a peculiar feeling in the pulse, which is apt to dissuade the practitioner from the employment of blood-letting. The pulse though small, will however be found more or less *incompressible*, and whilst blood is flowing from a vein, it acquires more and more volume, and often a very considerable quantity is abstracted before a fainting state supervenes.

"The abstraction of a quantity of blood ought to be had recourse to, before and after most surgical operations. When operations prove unsuccessful, I have always remarked that in by far the majority of cases, the patients die of inflammation of some internal organ. Bleeding therefore will, when early resorted to, and carried to a proper extent, have the effect of always checking any inflammatory disposition, and it will also be the means of securing the healing of the wound by adhesion, when such is desirable.

"The modes of abstracting blood for such purposes consist, either in taking away a quantity before the operation,—in allowing the divided vessels to bleed during the operation—or in employing venesection after the operation. Now all these *three* methods ought to be adopted separately or conjointly in particular cases.

"When an operation is to be performed on a plethoric subject, and an operation where little blood can be lost—more particularly in operations for *cataract*, in which no blood-vessel is ever divided,—then it is very judicious to bleed the patient on the morning of the operation; any future bleeding, of course, depending on the snbsequent symptoms.

"Bleeding, too, not only increases the action of the bowels, but it promotes the mercurial action. Dr. Fordyce used to say that in some cases of constipated bowels, we ought to open them with a lancet; and the late Mr. Gibson of Manchester was in the habit of bleeding largely when he was anxious to influence the system rapidly with mercury, and I have repeatedly observed how quickly affected was the system of those who at the same time were bled."

We are glad to find that this valuable little work has been republished in this country in the first number of Prof. Dunglison's excellent periodical—the American Medical Library and Intelligencer. We take this occasion of giving our earnest recommendation of this new and able semi-monthly "Record of Medical Science and Literature." We hope that the profession will give it their effective support, for it is, in every way, worthy of a wide and liberal patronage from the medical public.

